# Editorial: Mechanisms of cholinergic transmission in motivation and cognition

**DOI:** 10.3389/fnmol.2025.1586940

**Published:** 2025-03-26

**Authors:** T. Chase Francis, Anna M. Klawonn

**Affiliations:** ^1^Drug Discovery and Biomedical Sciences, College of Pharmacy, University of South Carolina, Columbia, SC, United States; ^2^Danish Institute of Translational Neuroscience (DANDRITE), Nordic EMBL Partnership for Molecular Medicine, Aarhus University, Aarhus, Denmark; ^3^Department of Biomedicine, Aarhus University, Aarhus, Denmark

**Keywords:** acetylcholine, motivation, reward, cognition, addiction, nicotinic receptor (nAChR), muscarinic receptor

Acetylcholine was one of the first fast neurotransmitters to be discovered and measured in synaptic recordings (Loewi and Navratil, 1926; Dale, 1914, 1934; For a historical overview see Tansey, 2006). Since then, researchers have found a central role for acetylcholine in numerous functions including attention, memory, motivation, and mood (Tobin, 2024; Cox and Witten, 2019; Picciotto et al., 2012). Disruption of cholinergic transmission has been found in various neuropathology, from Alzheimer's Disease to Addiction disorders (Zhang et al., 2024; Tobin, 2024; Williams and Adinoff, 2008).

For this Research Topic, we have selected 6 original research and review articles exploring the molecular mechanisms of cholinergic transmission in motivation, reward, and reinforcement learning. Including, novel studies on the role of distinct receptor populations and circuitry relevant for attention, reward and motivation (Fritz et al.; Braunscheidel et al.; Kim et al.; Berezovskaia et al.), as well as two reviews of the most relevant studies on cholinergic circuits and striatal cholinergic interneurons (Ratna and Francis; Runyon et al.).

All main cholinergic brain systems have been linked to reward related computations (Ruan et al., 2022). Cholinergic input to the motivation-associated mesolimbic dopamine system arises from distinct neural populations, including projections from two brainstem nuclei, the laterodorsal tegmental nucleus and the pedunculopontine nucleus (PPN), as well as a small population of interneurons in the striatum, comprising 1%−2% of all striatal neurons (Dautan et al., 2014; Oakman et al., 1995). Acetylcholine exerts its actions through two receptor families, which originate from distinct genes and are functionally classified by their pharmacological ligand selectivity: Nicotinic and muscarinic acetylcholine receptors.

Nicotinic acetylcholine receptors are fast-acting ligand-gated ion channels that facilitate cation influx upon acetylcholine binding, while Muscarinic receptors are slow-acting G-protein coupled receptors, mediating modulatory effects on target neurons (Tobin, 2024; Mihailescu and Drucker-Colin, 2000). In the mammalian central nervous system (CNS), restrictive or selective expression of these receptors in different brain regions dictate their function (Ahmed et al., 2019). The most widely expressed nicotinic receptor subtypes are the α7 homomeric and α4β2 heteromeric nAChRs (Hendrickson et al., 2013); while the muscarinic acetylcholine receptors consist of five subtypes (M1-M5), which all are widely expressed throughout the CNS (Tobin, 2024).

For decades, the muscarinic receptor family has been the focus of drug development for treating neuropathology, despite the important role of nicotinic receptors in cognitive and reward functions (Tobin, 2024). In this Research Topic, Braunscheidel et al. explored a novel positive allosteric modulator of α4β2 nicotinic receptors, SR9883, for general reward and nicotine reinforced behaviors. They found significant effects on nicotine reward suggesting that SR9883 may hold promise as a novel treatment of tobacco use disorder (Braunscheidel et al.).

In the research article, “*Nicotinic* α*7 receptors on cholinergic neurons in the striatum mediate cocaine reinforcement, but not food reward*,” Fritz et al. uncovered a new role for nicotinic α7 receptors on striatal cholinergic interneurons selective in regulating cocaine seeking and reward, but not natural food reward. These findings suggest that acetylcholine signaling through nicotinic α7 receptors regulate drug selective behaviors, linking nicotinic receptor signaling not only to nicotine reward but also to psychostimulant addiction (Fritz et al.).

Striatal cholinergic interneurons (CINs) are rare but exert widespread influence on striatal output by regulating dopamine and glutamate signaling (Cox and Witten, 2019; Picciotto et al., 2012). These interneurons exhibit tonic firing but can generate phasic responses to salient stimuli, contributing to processes such as learning, plasticity, and motor control (Zhang and Cragg, 2017). In the review article “*Extrinsic and intrinsic control of striatal cholinergic interneuron activity,”* Ratna and Francis explore the role of CINs, providing an overview of significant findings on their activity. They focus on intrinsic factors and neuromodulators that govern phasic CIN responses involved in learning and plasticity (Ratna and Francis).

Acetylcholine exerts neuromodulatory actions through muscarinic GPCRs on CINs, including the M4-receptors (Zhang et al., 2002; Caulfield, 1993). Berezovskaia et al. found that selective ablation of muscarinic M4 receptors from cholinergic neurons influences locomotor responses to cocaine and scopolamine in a sex-specific manner. The study points out important discrepancies between experimental findings, potentially arising from distinct mouse-lines and gender differences, relevant for both study design considerations and interpretation within the field (Berezovskaia et al.).

In the paper “*Distinct cholinergic circuits underlie discrete effects of reward on attention*,” Runyon et al. review the role of cholinergic circuits in reward and cognition, further examining the contexts in which attention and reward computations interact, to propose two discrete neural circuits responsible for stimulus-reward associations (Runyon et al.).

Kim et al. explored the role of PPN cholinergic neurons for updating action-outcome expectations in a reward reversal learning paradigm using a Designer Receptors Exclusively Activated by Designer Drugs (DREADDs) strategy in rats (Roth, 2016). Collectively, their findings suggest that cholinergic PPN neurons are essential for flexible update of behavioral strategies for maximizing reward (Kim et al.).

The articles of our Research Topic provide insight in the most recent findings on cholinergic receptor and circuit dynamics in motivation and cognition. We hope that this Research Topic will contribute to the understanding and furthering of scientific progress in the field; and that it will be as interesting to read as it was for us to compose it.
